# Burn wounds after electrical injury in a bathtub: a case report

**DOI:** 10.1186/s13256-019-2231-4

**Published:** 2019-09-26

**Authors:** Sem F. Hardon, Pieter J. Haasnoot, Annebeth Meij-de Vries

**Affiliations:** 1 Department of Surgery, Amsterdam UMC - VU University Medical Center, Room ZH 7F 020, P.O. Box 7057, 1007 MB Amsterdam, The Netherlands; 2 Department of Surgery, Red Cross Hospital, Beverwijk, The Netherlands; 3 Burn Center, Red Cross Hospital, Beverwijk, The Netherlands

**Keywords:** Burn wounds, Electrical injury, Smartphone, Bathtub

## Abstract

**Background:**

Increased smartphone use among minors makes our population more prone to electrical injury. Despite regulations on electrical home safety standards, smartphones and chargers still pose a risk for severe injury among users.

**Case presentation:**

We present a case of a patient with low-voltage electrical burns due to smartphone use in a bathtub. The 13-year-old Caucasian patient was using a smartphone plugged into the electrical grid while taking a bath. We report the burns and their treatment. We discuss the likely burn mechanism.

**Conclusions:**

Burn wounds after electrical injury due to smartphone use are rare. The presented case shows the danger of smartphone use in bathtubs.

## Background

Risks of electrical devices in household use are underestimated. Although products are thoroughly tested and certified, they pose a great danger if used inappropriately. Electrical injuries can be caused by low-voltage electric current (50–1000 V) and high-voltage electric current (> 1000 V). Electrical injury in children usually occurs at home and is most commonly caused by low-voltage current [1–3]. Most electrical injuries in adults are work-related injuries and are a repeatedly described cause of occupation-related death [2, 4]. A severe electrical burn injury can cause destructive injury with high morbidity, lifelong scars, and even death [5, 6].

In children, most injuries occur in the home environment [6–8]. Despite regulations on electrical home safety standards, smartphones and chargers still pose a risk for severe injury among users. Few articles have been published concerning these health risks.

Recently, media reports have shown that accidents as a result of smartphone use occur. Even smartphone injuries leading to death have been reported [9–13]. Increased smartphone use among minors might be the reason our population is more prone to these risks. The aim of this case report is to raise awareness of this topic and to evaluate considerations for treatment.

## Case presentation

A 13-year-old Caucasian girl with a deep burn injury was referred to our burn center. She had no medical history. She had held a charging smartphone in her right hand while taking a bath. After hearing a loud scream, the mother of the patient disconnected the charger from the sparkplug and took her daughter out of the bath.

According to the mother, the patient was briefly unconscious and showed muscle contractions. After receiving a precordial thump, the patient became responsive again.

The patient was evaluated according to the Advanced Trauma Life Support guidelines and was founded to be stable both with regard to respiration and hemodynamically.

**Fig. 1:**
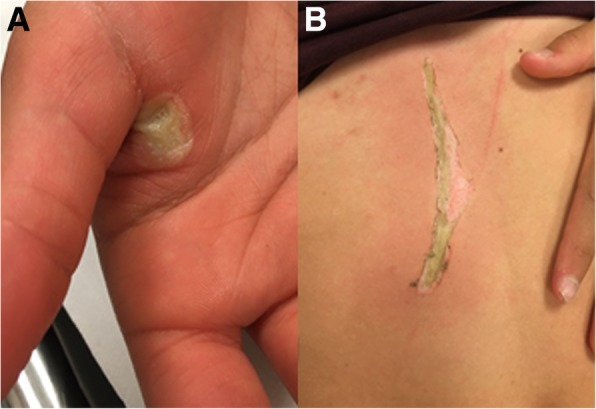
**a** Right hand with burn wound between thumb and index finger on the palmar side. **b** Stripe-shaped burn over the abdomen near the epigastric region

Further physical examination showed two deep burns: (1) a circumscribed, oval-shaped lesion of approximately 1 × 1 cm, with a central zone of pallor on the palmar side of the hand between thumb and index finger, and (2) a stripe-shaped laceration of the skin of approximately 1 × 12 cm, on the abdomen, near the epigastric region, surrounded by a zone of hyperemia (Fig. 1). The patient’s total body surface area burned was less than 0.5%.

The patient’s serum creatinine kinase (CK) level was 1294 U/L, which is severely elevated. No abnormalities were seen in electrocardiograms. The patient was admitted to the pediatric ward for observation and a tertiary survey. The day after admission, her CK level was slightly elevated to 1400 U/L. Her urine was tested for myoglobinuria but showed no signs of rhabdomyolysis. Furthermore, the patient had no complaints or other abnormalities at the tertiary survey and was therefore discharged to home at on postburn day 2.

Owing to amnesia, the patient failed to give a detailed reconstruction of the moment of injury. Muscle twitches, the severity of the burns, and muscle decay (elevated CK level) indicated that an alternating current with 240 V caused the electrical injury, which is the standard voltage in the Netherlands. There has probably been direct conductance between main voltages from the spark plug to a grounded element in the bathtub, such as the drain. Another explanation is that the current flowed over the outer side of the charging cable, which was moist, to the patient. Moist skin is more vulnerable to electrocution injury because of decreased resistance.

**Fig. 2:**
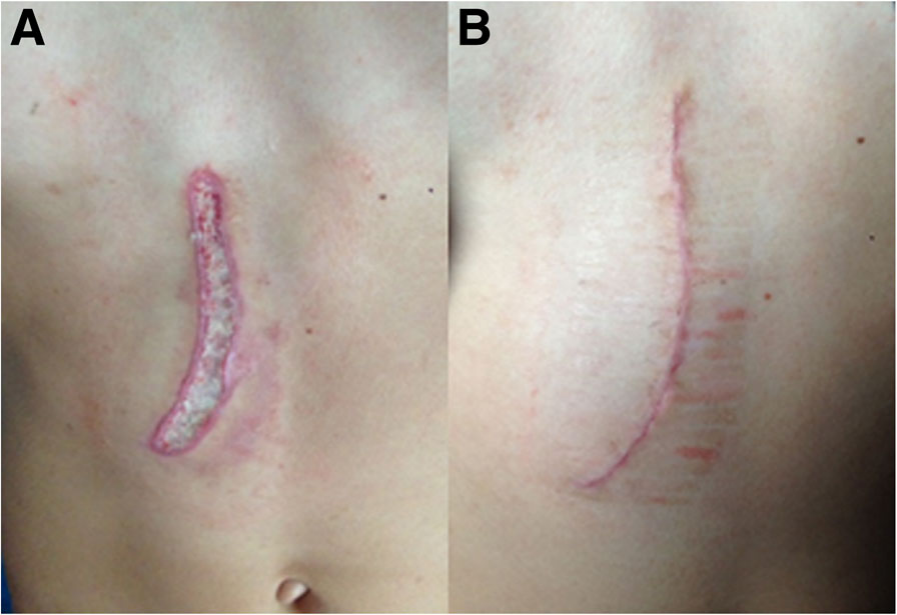
**a** The burn wound over the abdomen after 21 days. **b** Scar 10 days after surgical resection, transposition, and wound closure

Initially, the burns were treated conservatively with silver sulfadiazine cream, which was altered to fusidic acid cream after 1 week. This treatment was adequate for the burn wound on the hand because this was healing. After 21 days, the abdominal burn had healed insufficiently. Therefore, surgical resection and transposition of the skin were performed (Fig. 2). This resulted in satisfactory healing with little scarring.

## Discussion and conclusions

This case report describes the effect of electrical injury resulting in deep burn wounds. In children, most electrical injuries occur in the home environment and are caused by low-voltage electric current. Children are prone to this injury while playing with faulty electrical devices or misusing them [14]. It is clear that smartphone use among children has become increasingly popular. The prevalence of electrocution due to misuse of smartphones can probably be explained by both lack of supervision and lack of awareness among users.

The severity of burn injury due to electrocution depends on the factors of electricity and the human body [15]. The type and intensity (that is, voltage) of current, the location and duration of contact on the body, and the organs are affected are factors [14, 16]. In addition, human and environmental factors such as physical and nutritional state and humidity can influence the resistance of the skin. Tissues such as fat, bone, and skin have relatively high resistance to electricity and therefore tend to increase in temperature and coagulate [2, 5]. The impact of the electrocution can increase if the skin is wet or if the electrocution occurs in a humid environment [2, 15]. This can be explained by Ohm’s law, in which the current is inversely proportional to resistance. All clinicians should be aware of these factors when reconstructing the mechanism of injury. Moist skin may receive a less superficial thermal injury but allows more current into the body. Therefore, the risk of damage to internal organs increases [6].

Serum CK level and signs of myoglobinuria should be investigated directly after presentation at the emergency department and can indicate internal damage. Limitations on the specificity and sensitivity of the tests as mentioned above, as well as serum myoglobin, should be considered before using these tests to investigate muscle ischemia and cell breakdown [17]. Also, serum levels of lactate dehydrogenase, transaminases, and potassium and blood level of phosphorus could indicate risk of renal morbidity and should be considered for testing [17]. Although our patient showed no signs of myoglobinuria, we did not rule out mild rhabdomyolysis, which was most likely the cause of her elevated CK level [18].

The severity of burn injury caused by an electric current is dependent on multiple factors, which should be taken into account when diagnosing and treating patients with electrical injuries. We emphasize that many injuries could be avoided by proper education and precautionary measures, which often involve a commonsense approach [6, 8]. However, prevention strategies should be used to educate parents and schools of young children regarding the safe use of portable household devices and electrical cords. Older school-aged children should be educated at school [19, 20].

If household electrical devices are used in bathrooms, users must be aware of safety hazards, and the devices must comply with safety regulation standards. Nonetheless, electrical appliances or cords should never be plugged in near water and should never be in contact with a wet environment [21].

## Data Availability

Not applicable.

## Abbreviation

CK: Creatine kinase

## References

[1] Mashreky SR, Rahman A, Khan TF, Svanstrom L, Rahman F. Epidemiology of childhood electrocution in Bangladesh: findings of national injury survey. Burns. 2010;36(7):1092–5. 10.1016/j.burns.2010.01.008 20395057

[2] Spies C, Trohman RG. Narrative review: electrocution and life-threatening electrical injuries. Ann Intern Med. 2006;145(7):531–7. 10.7326/0003-4819-145-7-200610030-00011 17015871

[3] Duci SB, Arifi HM, Ahmeti HR, Selmani ME, Buja ZA, Gashi MM, *et al.* Electrical burn injuries of 246 patients treated at the University Clinical Center of Kosovo during the period 2005-2010. Eur J Trauma Emerg Surg. 2014;40(6):679–85. 10.1007/s00068-014-0379-6 26814782

[4] Arnoldo BD, Purdue GF, Kowalske K, Helm PA, Burris A, Hunt JL. Electrical injuries: a 20-year review. J Burn Care Rehabil. 2004;25(6):479–84. 10.1097/01.bcr.0000144536.22284.5c 15534455

[5] Nagesh KR, Kanchan T, Rastogi P, Arun M. Arcing injuries in a fatal electrocution. Am J Forensic Med Pathol. 2009;30(2):183–5. 10.1097/PAF.0b013e3181875ad9 19465814

[6] Glatstein MM, Ayalon I, Miller E, Scolnik D. Pediatric electrical burn injuries: experience of a large tertiary care hospital and a review of electrical injury. Pediatr Emerg Care. 2013;29(6):737–40. 10.1097/PEC.0b013e318294dd64 23714758

[7] Faruque AV, Mateen Khan MA. Unintentional injuries in children: are our homes safe? J Coll Physicians Surg Pak. 2016;26(5):445–6. 27225158

[8] Roberts S, Meltzer JA. An evidence-based approach to electrical injuries in children. Pediatr Emerg Med Pract. 2013;10(9):1–16. quiz 16–7 24191428

[9] Moscow woman electrocuted after dropping iPhone 4 in bathtub. Moscow Times. 10 Feb 2015. https://themoscowtimes.com/articles/moscow-woman-electrocuted-after-dropping-iphone-4-in-bathtub-43732. Accessed 1 March 2019.

[10] Levin P. Teen electrocuted after playing on phone in bathtub. 8 July 2017. https://edition.cnn.com/2017/07/18/health/teen-bathtub-electrocuted-text-trnd/index.html. Accessed 1 March 2019.

[11] Man dies charging iPhone while in the bath. 17 Mar 2017. https://www.bbc.com/news/uk-39307418. Accessed 1 March 2019.

[12] McClaughlin K. Pregnant woman is electrocuted while charging her phone as she used it in the bath two weeks before she was due to give birth. Dly Mail. 2 Jan 2018. http://www.dailymail.co.uk/news/article-5229185/Woman-electrocuted-charging-phone-near-bathtub.html. Accessed 1 March 2019.

[13] Stewart W. Russian schoolgirl, 12, is ‘electrocuted in the bath’ after dropping her mobile in the water while charging it. Dly Mail. 2 Feb 2018. http://www.dailymail.co.uk/news/article-5343581/Russian-schoolgirl-12-dies-dropping-phone-bath.html. Accessed 1 March 2019.

[14] Wick R, Gilbert JD, Simpson E, Byard RW. Fatal electrocution in adults—a 30-year study. Med Sci Law. 2006;46(2):166–72. 10.1258/rsmmsl.46.2.166 16683472

[15] Zhang P, Cai S. Study on electrocution death by low-voltage. Forensic Sci Int. 1995;76(2):115–9. 10.1016/0379-0738(95)01804-2 8566912

[16] Ungureanu M. Electrocutions – treatment strategy (case presentation). J Med Life. 2014;7(4):623–6. PMC4316151 25729443

[17] Keltz E, Khan FY, Mann G. Rhabdomyolysis: the role of diagnostic and prognostic factors. Muscles Ligaments Tendons J. 2013;3(4):303–12. PMC3940504 24596694

[18] Huerta-Alardin AL, Varon J, Marik PE. Bench-to-bedside review: Rhabdomyolysis – an overview for clinicians. Crit Care. 2005;9(2):158–69. 10.1186/cc2978 PMC1175909 15774072

[19] Rabban JT, Blair JA, Rosen CL, Adler JN, Sheridan RL. Mechanisms of pediatric electrical injury: new implications for product safety and injury prevention. Arch Pediatr Adolesc Med. 1997;151(7):696–700. 10.1001/archpedi.1997.02170440058010 9232044

[20] Johnson EL, Maguire S, Hollen LI, Nuttall D, Rea D, Kemp AM. Agents, mechanisms and clinical features of non-scald burns in children: a prospective UK study. Burns. 2017;43(6):1218–26. 10.1016/j.burns.2017.01.036 28645715

[21] Clark AT, Wolf S. Electrical Injury. JAMA. 2017;318(12):1198. 10.1001/jama.2017.11156 28973623

